# Physician Attitude toward Their Attires and Laundering Habit Changes during the COVID-19: A Cross-Sectional Survey in a Tertiary Care Center

**DOI:** 10.1055/s-0043-1770935

**Published:** 2023-08-04

**Authors:** Reema Alwabel, Bushra Alasmari, Aljawhara Alabdulkarim, Yusra Chachar, Hamdan A. Jahdali, Laila Layqah, Salim Baharoon

**Affiliations:** 1 College of Medicine, King Saud bin Abdulaziz University for Health Sciences, Riyadh, Saudi Arabia; 2 King Abdullah International Medical Research Center, Riyadh, Saudi Arabia; 3 Ministry of National Guard-Health Affairs, Riyadh, Saudi Arabia; 4 College of Science and Health Professions, King Saud bin Abdulaziz University for Health Sciences, Riyadh, Saudi Arabia; 5 Department of Medicine, King Abdulaziz Medical City, Riyadh, Saudi Arabia; 6 King Saud bin Abdul-Aziz University for Health Sciences, Riyadh, Saudi Arabia; 7 Research Offices Department, King Abdullah International Medical Research Center, Riyadh, Saudi Arabia.

**Keywords:** physician attire, cleaning practice, patient safety

## Abstract

**Background** Patient safety is of utmost importance and every effort is to be made to reduce the risk of hospital-acquired infection. Contaminated attire is proposed as a mode of hospital infections spread. This study aims to assess the laundering habits, the perception of healthcare workers toward the contamination of their attire, and the effect of coronavirus disease 2019 (COVID-19) pandemic on their cleaning practices in non-operative settings.

**Methods** This is a cross-sectional study conducted using a self-administered questionnaire which was distributed among physicians at King Abdul-Aziz Medical city, Riyadh. The questionnaire queried the physicians about their laundering habits, knowledge toward their attire, and the difference in cleaning practices after the emergence of COVID-19.

**Results** Out of 220 questionnaires distributed, 192 physicians responded. Majority of physicians were male (54%) and were in the 20 to 30 age group. Female gender was significantly associated with the frequency of uniform washing ( *p-* value < 0.0001) and place of cleaning (home vs. outside home) ( *p-* value <0.0001). Physicians in intensive care were more likely to take off their uniforms daily before leaving the hospital compared to others ( *p-* value of 0.018). Most physicians did not prefer to use the hospital laundry system for cleaning their uniforms but consultants were the most to use it. COVID-19 pandemic led to changes in washing habits in 108 physicians (60%).

**Conclusion** Majority of physicians accepted washing their uniforms multiple times per week and their washing habits increased during the COVID-19 pandemic. Female gender and younger physician both were associated with increasing washing habits.

## Introduction

Healthcare-associated infection (HAI) has always been a major public health concern. HAI is defined as an infection that occurs in patients during their hospital stay but was not acquired before or at the time of admission. 1 HAI is a significant problem for patient safety, as it results in prolonged hospital stay, persistent antimicrobial resistance, and a financial burden on healthcare systems. 2 The consequences of these infections have led to the development and implementation of a proper approach to infection control and prevention systems. 3 Since pathogens associated with HAI commonly spread through direct and indirect contact, standard infection control practices have centered on hand hygiene and using personal protective equipment (PPEs) among other measures. Over the recent decades, however, contaminated hospital environments are being increasingly implicated as a point source in the transmission of such infection. 1

Recently, there has been a concern about whether healthcare workers' (HCWs) attire is a potential vehicle of transmitting pathogenic organisms in hospital setting. 4 5 Accidental exposure to body fluid and blood might be inevitable especially in nonoperative setting as it is less supervised when compared to operative setting, in addition to the inadequate adherence to PPE and standard infection control precautions. 6 Moreover, it is not uncommon for some HCWs to wear hospital uniform when visiting nonhealthcare-related properties near to the health institution, or socialize outside the institution while still wearing their hospital uniforms, which carries the risk of transferring pathogens, from the hospital and import other microorganisms from the environment. 7 8 Several studies have examined the contamination of white coats and scrubs worn by HCWs and found evidence of pathogenic organisms including *Clostridium difficile* and drug-resistant organisms like methicillin-resistant Staphylococcus aureus (MRSA). 7 9 One recent systematic review has concluded that HCWs attire could be a potential source of transmission of pathogenic organisms in the hospital setting, although a direct link is yet to be determined. 4

When considering the risk of spreading infection due to the contaminated attire, it is important to look into the laundering habits of hospital uniforms as it has a major impact on the rate of contamination with pathogenic bacteria such as MRSA. A few studies have showed that uniforms that were washed daily had the lowest rate of contamination. 5 10 In addition, this topic is of particular importance nowadays with the emergence of COVID-19 pandemic, as evidence suggests that severe acute respiratory syndrome coronavirus 2 remains infectious on cotton for 5 minutes to 24 hours, with variability attributed to the initial titter, and for about 48 hours on disposable gowns. 11 12

This study aimed to assess the laundering habits and perception of HCWs toward the contamination of their attire in nonoperative settings. Furthermore, the effect of COVID19 pandemic on their cleaning practices was investigated as well. In our hospital, there is no section in the infection control manual dedicated to employee cloth washing instruction during the COVID 19 pandemic.

## Methods

### Study Design and Settings

This is a cross-sectional study conducted using a self-administered questionnaire in the period from April 1 to August 30, 2021. The questionnaire was developed by the authors and was validated by preliminary pilot testing for consistency. It was reviewed by multiple experts in the field of medicine.

The questionnaire is composed of three sections. The first section assessed physicians' demographic data that included all the variables. The second section contained five close-ended questions regarding their practice of washing uniforms, and it was answered only by those who consider white coats or scrubs as their usual uniform. The third section evaluated their knowledge regarding their uniform.

The questionnaire was distributed in three departments: intensive care, internal medicine, and general pediatrics. Participants included consultants, fellows, residents, interns, and medical students. Participants with missing data were excluded.

King Abdulaziz Medical City in Riyadh is a tertiary healthcare center of more than 1000-bed capacity. It is one of the major centers in Saudi Arabia and gets referral from across the whole country. The main hospital has a laundry service that was open 24 hours, 7 days a week. New hospital uniforms were given to HCWs at least annually and sometime every 6 months.

### Data Analysis

The data was entered in Microsoft Excel and then exported to Statistical Package for Social Sciences (SPSS) version 25 for analysis. All variables were qualitative and were reported as frequencies and percentages. The relationships between variables were analyzed using the chi-squared test or Fisher's exact test as appropriate. All *p* -values were considered significant at 5% level of significance. The recruitment period was from April 1, to August 30, 2021.

## Results

### Demographic Characteristics

Among the 220 medical professionals approached to complete the survey, 192 participants responded (response rate of 87%). Out of those, 104 (54%) were males, and the majority were in the 20 to 30 age group ( *n* = 132, 69%). One-hundred six (58%) participants have been in the medical field for 1 to 5 years; 76 (40%) were residents and only 35 (19%) were assistant consultant/consultant. Other characteristics of the respondents are shown in Table 1 .

**Table 1:** Sociodemographic characteristics of study participants ( *n* = 192)

Variables	*n* (%)
***Gender*** • Male • Female	104(54) 88 (46)
***Age*** • 20–30 • 31–40 • 41–65	132 (69) 41(21) 19 (10)
**Professional experience** • 1–5 years • 6–10 years • >10 years	106 (55) 48 (25) 38 (20)
**Professional category** • Student • Intern • Resident • Fellow • Assistant consultants • Consultant	34 (18) 18 (9) 76 (40) 29 (15) 11(6) 24 (13)
**Departments** • Internal medicine • Intensive care • General pediatrics	103 (54) 34 (18) 55 (29)
**Type of the usual daily clinical uniform** • White coat and scrub • Scrub only • White coat with everyday wearing/suit • Everyday wearing/suit • Thoub and Shumagh/Gutra	118 (62) 16 (8) 50 (26) 4 (2) 4 (2)

### Cleaning Practices and Attitude

**Fig. 1:**
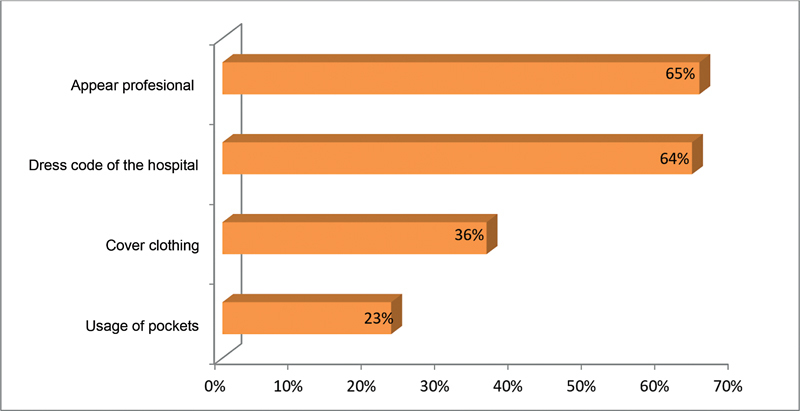
Reasons for wearing white coat.

The majority of respondent ( *n* = 169, 88%) reported wearing a white coat to work. Out of those, 125 (65%) justified that the reason was to appear professional, 124 (64%) reported it is the dress code of the hospital, 70 (36%) used white coat to cover clothing, and 45 (23%) wore white coat for usage of pockets ( Fig. 1 ). On evaluation of the practice of washing uniforms, 81 (42%) washed their scrub every day and 66 (34%) reported that they wash their white coat every 2 to 4 days ( Table 2A ). One-hundred fifteen (60%) reported a change in their washing habits during COVID-19 ( Table 2B ).

**Table 2A:** Uniform cleaning practices results among healthcare workers

Variables	*n* (%)
**Time of taking off uniform** • Before leaving the hospital • Arrive at home • Later in the day	33 (17) 156 (81) 3 (2)
**Type of uniform cleaning** • Home washing • Hospital laundry • Outside laundering service	145 (76) 11 (6) 36 (19)
**Do you separate your hospital uniform/white coat from other household laundry** • Yes • No	109 (57) 83 (43)
**Which of laundering techniques do you follow? (multiple answers can be chosen)** ***n*** **= 282** • Using hot water • Using Ironing after washing • Using tumble drying • Using regular washing machine • I don't know	70 (25) 77 (27) 49 (17) 14 (5) 72 (26)
**Frequency of white coat washing** • Everyday • 2–4 times a week • Weekly • Every 2 weeks	60 (31) 73 (38) 53 (28) 6 (3)
**Frequency of scrub washing** • Everyday • 2–4 times a week • Weekly • Every 2 weeks	81 (42) 66 (34) 32 (16.7) 13 (6.7)

**Table 2B:** Percentage distribution of healthcare workers according to specific attitude items

Variables	*n* (%)
Change of washing habits after COVID-19 • Yes • No	115 (60) 77 (40)
Do you believe that the uniform is clean if it has no visible stain? • Yes • No	29 (15) 163 (85)
Do you think that the uniform can carry pathogens? • Yes • No	188 (98) 4 (2)
Do you think that uniform can be a source of infection? • Yes • No	186 (97) 6 (3)
Did you take an infection control course? • Yes • No	142 (74) 50 (26)

Abbreviation: COVID-19, coronavirus disease 2019.

### Association of Demographic Characteristics with the Practice Regarding Uniforms

We measured the association between the frequency of washing white coats and scrubs with several variables including gender, professional category, and department. Gender was significantly associated with the frequency of washing attire. As illustrated in Table 3A , 39 (44%) females reported that they wash their white coats every day compared to only 21 (20%) males who wash theirs every day ( *p* -value < 0.0001). Overall, the majority of females wash their white coats every day or 2 to 4 times a week (44, 42%), respectively, whereas most of the males reported washing their white coats either once weekly or two to four times a week (39, 35%), respectively. Similarly, female gender was associated with more daily washing of their scrubs (52.3% female vs. 33.6% male) or two to four times a week (31% female vs. 37% male). Junior physicians (students, interns, and residents) tend to do more frequently washing of their white coats and scrubs as compared to more senior physicians (fellow and consultants).

**Table 3A:** Association of demographic characteristics with the practice regarding uniforms

		Frequency of white coat washing					Frequency of scrub washing				
	Variable	Everyday, *n* (%)	2–4 X a week, *n* (%)	Weekly, *n* (%)	Every 2 weeks, *n* (%)	*p-* Value	Everyday, *n* (%)	2–4 X a week, *n* (%)	Weekly, *n* (%)	Every 2 weeks, *n* (%)	*p* -Value
Gender	Male (104)	21 (20)	36 (35)	41 (39)	6 (6)	< 0.001	35 (33.6)	39 (37.5)	23 (22)	7 (6.7)	0.011
	Female (88)	39 (44)	37 (42)	12 (14)	0		46 (52.3)	27 (30.6)	9 (10.2)	6 (6.8)	
Professional category	Student	15 (44)	14 (41)	5 (14)	0	0.022	17 (50)	14 (41)	3 (9)	0	< 0.001
	Intern	6 (33)	10 (55)	2 (11)	0		9 (50)	6 (33)	2 (11)	1(6)	
	Resident	23 (30)	28 (37)	23 (30)	2 (3)		32 (42)	26 (34)	16 (21)	2 (3)	
	Fellow	5 (17)	15 (52)	9 (31)	0		11 (38)	15 (51.7)	1 (3.4)	2(6.9)	
	Assistant consultants	3 (27)	3 (27)	4 (36)	1 (9)		5(45.5)	1 (9)	3 (27)	2 (18)	
	Consultant	8 (33)	3 (13)	10 (42)	3 (13)		7 (29)	4 (16.7)	7 (29)	6 (25)	
Departments	Internal medicine	31 (30)	43 (42)	28 (27)	1 (1)	0.109	46 (44.5)	36 (35)	18 (17.5)	3 (3)	0.034
	Intensive care	13 (38)	11 (32)	10 (29)	0		15 (44.2)	11 (32.3)	5 (14.7)	3(8.8)	
	General pediatrics	16 (29)	19 (35)	15 (27)	5 (9)		20 (36.4)	19 (34.5)	9 (16.4)	7 (12.7)	

Majority of physicians (81%) take off their uniforms at home rather than the hospital. The place of taking uniforms off was significantly associated with the department of the participant with a ( *p* -value = 0.018), reporting that intensive care unit (ICU) physicians were more likely to take their white coats off before leaving the hospital compared to internal medicine and pediatrics (35, 14, and 13%, respectively; Table 3B ).

**Table 3B:** Association of demographic characteristics with the practice regarding uniforms

		Place of taking off uniform				Place of uniform cleaning			
	Variable	Before leaving the hospital	Arrive at home	Later in the day	*p* -Value	Home washing	Hospital laundry	Outside laundering service	*p* -Value
**Gender**	Male	21 (20)	82 (79)	1 (1)	0.391	65 (63)	10 (10)	29 (28)	< 0.001
	Female	12 (14)	74 (84)	2 (2)		80 (91)	1 (1)	7 (8)	
**Professional category**	Student	4 (12)	29 (85)	1 (3)	0.117	30 (88)	0	4 (12)	< 0.001
	Intern	0	17 (94)	1 (6)		15 (83)	0	3 (17)	
	Resident	11 (15)	65 (85)	0		56 (74)	0	20 (26)	
	Fellow	9 (31)	20 (69)	0		23 (79)	1 (3.4)	5 (17)	
	Assistant consultants	3 (27)	8 (72)	0		9 (82)	1 (9)	1 (9)	
	Consultant	6 (25)	17 (71)	1 (4)		12 (50)	9 (38)	3 (13)	
**Departments**	Internal medicine	14 (14)	86 (84)	3 (3)	0.018	75 (73)	4 (4)	24 (23)	0.353
	Intensive care	12 (35)	22 (65)	0		27 (79)	2 (6)	5 (15)	
	General pediatrics	7 (13)	48 (87)	0		43 (78)	5 (9)	7 (13)	

However, about are uniforms cleaned is significantly associated with the gender and seniority of participants. Female physicians prefer to wash their uniforms at home (91%) as opposed to 63% of male physicians ( *p* < 0.0001). Male (28%) use outside laundry service compared to (8%) females. In addition, only males (10%) reported using hospital laundry ( Table 3B ). Consultants were the least group to wash their uniforms at home (50%) and more likely to use the hospital laundry system ( Table 3B ).

## Discussion

Our study showed that about most physicians wash their uniforms very frequently. We also found that over half of the study subjects noted a change in their washing habits after the emergence of COVID-19 pandemic, with the majority reporting an increase in washing frequency compared to before. The average days between washing white coats were 4 days. Some studies reported the average days between washing white coats around 12 to 14 days. 13 14 We believe that having our study taken place after the COVID-19 pandemic could account for this difference, as many people became more aware of the role of attire and inanimate objects in cross-contamination and, consequently, apply more meticulous hygienic practices. In general, white coats are washed less frequently than scrubs.

This is in line with findings from Munoz-Price et al *,* which also found an association between bacterial contamination of white coats and contaminated HCW's hands but could not find the same association with contamination of scrubs. 14 Although the reason for this paradoxical behavior is unclear, it could be due to HCW's perception of scrubs being more spoiled as they come in direct contact with their own skin and body fluids.

Our results showed that a minority of males washed the white coat every day compared to females. This might be explained by the fact that females are more likely to care about cleanliness and hygiene. This is in line with Neves et al finding which showed that males changed their white coat less frequently and were keen to change it only if it was visibly soiled. 15 Wiener-Well et al have also found that infrequent changing associated with higher contamination rate of resistant micro-organisms and was higher in males. 16 These findings may be allegedly explained by gender character where females were more organized and tidier or a reflection of a more emotional component of fear of spread to family members or could reflect a different understanding of how disease can spread.

Multiple studies have showed that taking the hospital uniform off, particularly white coats, before leaving the hospital is an essential part of infection control practices. Mwamungule et al evaluated the white coats usage/handling practices and bacterial contamination among health workers. Overall white coats have high contamination rate and those taken after work and stored at home have higher contamination rate than those left at hospital. This is likely because those left at hospital will likely get cleaned more frequently as physician may sense they are more likely contaminated. 17 In our study, ICU physicians were more likely to take their white coats off before leaving the hospital compared to the other departments. According to Paul et al *,* there was a significant difference in scores of knowledge on HAIs and infection control practices across different departments, in which the ICU scored the maximum. 18 This difference in knowledge and attitude among different departments, resulting in ICU physicians being more knowledgeable, could be explained by the fact that ICU is particularly considered a great possibility for the occurrence of cross contamination between ICU environment and HCWs. 19

Overall, the majority of our study population reported using home washing rather than hospital laundry or outside laundry services. This is in contrast to findings of Olvera-Lopez et al, as they reported more than half of their study population used hospital laundry. 13 They further suggested that use of hospital laundry service could explain the long median time between washing white coats in their study, due to its tight schedule. As such, the home washing predominance noted in our study could account for our study participants' frequent white coat washing relative to that cited in the literature. We also found that males were significantly more likely to use outside laundry services and hospital services than females, which could be another explanation for females washing their coats more frequently, as home washing is more feasible to perform on a nearly daily basis.

Unfortunately, our study did not examine the frequency of exposure to other potentially infectious material or other confounding factors that could affect the washing habits of the participants like workloads, the cost of washing uniforms and the level of workload. The majority of admissions during the study period were due to COVID-19 infection and although workloads were not detailed, but we did specify the job title. Workloads during pandemic crises were very high and but still closely equally across similar groups. Being conducted in a tertiary care hospital where participants are all well paid and with a free laundering facility available for all employees also provide similar confounding across all groups.

## Conclusion and Recommendations

This study highlighted the attitude of HCWs toward their attire. Although there are no established guidelines specific to laundering HCWs' attire, the general consensus is to wash white coats and scrubs at least once to twice weekly or whenever visibly soiled. Our study found that physicians wash their scrubs slightly more frequently than their white coats. In addition, most of the participants reported a change in their laundering habits after the emergence of COVID-19. Future local studies determining the effect of laundering habits on the rate of contamination of hospital uniforms are recommended.

## Limitations

Our study has several limitations. Our sample size was small, from a single center and did not include all HCWs from other departments. The findings may not be applicable in other geographical areas in Saudi Arabia or other countries. In this study, more residents filled the survey compared to consultants as they were more likely to be present at the department and had time. The convenient sampling technique could cause selection bias. The study was conducted using a self-administrated questionnaire that may be a source of bias and may not reflect the actual daily behavior.

Finally, the study design is cross-sectional that examined the association between multiple variables; however, this cannot establish a causal relationship and whether HCW's attire can transmit infection remains unanswered.
